# Influence of naps on sedentary time and physical activity in early childhood

**DOI:** 10.1038/s41598-022-25628-x

**Published:** 2022-12-08

**Authors:** Christine W. St. Laurent, Sanna Lokhandwala, Tamara Allard, Angela Ji, Tracy Riggins, Rebecca M. C. Spencer

**Affiliations:** 1 Present Address: Department of Psychological Sciences, University of Massachusetts Amherst, 135 Hicks Way, Tobin Hall, Amherst, MA 01003 USA; 2 Department of Psychology, University of Maryland College Park, Biology/Psychology Building, 4094 Campus Dr., College Park, MD 20742 USA; 3 Institute for Applied Life Sciences, University of Massachusetts Amherst, 135 Hicks Way, Tobin Hall, Amherst, MA 01003 USA; 4 Present Address: Department of Graduate Psychology, James Madison University, Harrisonburg, USA

**Keywords:** Lifestyle modification, Paediatric research

## Abstract

The objective was to determine if, in preschool-aged children, (1) nap habituality is associated with sedentary time and physical activity (movement behaviors), (2) nap physiology is associated with movement behaviors, and (3) if missing a nap, compared to taking a nap, affects movement behaviors on the same day and subsequent day. A within-subjects (44 children; 4.2 ± 0.6 years; 55.6% female), at-home study examined two experimental conditions (one afternoon each of nap- and wake-promotion with order counterbalanced) one week apart. Movement behaviors were derived from wrist-worn actigraphy (12.1 ± 3.1 days). Average movement behaviors were calculated from the overall study period with experimental days excluded**.** Movement behaviors were also extracted for the same day and the subsequent day of the two experimental conditions. Polysomnography was recorded during the nap-promoted condition. Children were classified as non-, intermediate-, or habitual-nappers. Although average movement behaviors were different between nap habituality groups, differences were not significant. There were no associations between movement behaviors and nap sleep stages, and no effects for nap condition or condition by nap habituality on same or next day movement behaviors. Findings do not suggest that naps and movement behaviors are related in children. Although a single missed nap was not detrimental to same or next day movement behaviors, future studies should explore effects of multiple days of subsequent nap restriction to examine potential cumulative effects.

## Introduction

Higher levels of physical activity and lower time spent sedentary are beneficial for cardiometabolic health and fitness, bone and skeletal health, motor and cognitive development, and psychosocial health in early childhood (i.e., 5 years and younger)^1–4^. However, physical activity and sedentary time—referred to here as movement behaviors—vary considerably during these early years. For example, in one review of objectively-measured behaviors (e.g., accelerometry-derived) in preschool children, moderate- to vigorous-intensity physical activity (MVPA) ranged from 2 to 41% and sedentary time ranged from 34 to 94%^5^. Given that early childhood represents a time when healthy behavior practices are introduced and set the stage for future activity levels across childhood^6^ and even into adulthood^7^, it is important to understand what influences and cultivates these behaviors.

While several correlates and determinants of movement behaviors have been explored in young children—such as demographic, physical health, social/cultural, and environmental factors^8–10^ –relations between sleep and movement behaviors have only recently been considered. Although low sedentary time and high physical activity have been beneficially associated with sleep in adults^11–13^, two recent reviews concluded that among young children, evidence for a sleep benefit on sedentary time and physical activity is generally low^14,15^. However, studies in early childhood have primarily focused on overnight sleep using observational designs. Although daytime sleep (i.e., naps) is a common component of overall 24-h sleep in early childhood^16,17^ the influence of nap sleep on movement behaviors in young children is unclear.

Within early childhood, the preschool years (i.e., ~ 3–5 years) represent a transitional period for sleep behaviors, as children shift from biphasic sleep (one nap and one overnight sleep bout per 24-h period) to monophasic sleep (a single overnight sleep bout per 24-h period)^16,17^. This transition is not homogenous across children and some researchers have posited that this transition reflects a developmental milestone^18^, reflecting critical periods of brain development^19,20^. According to a recent review, only a small percentage of children under 2 years ceased napping, but by 5 years, the majority of children no longer napped^21^. However, there was large variation in napping rates and the reason for napping could take different forms. While some preschool children may still have a physiologic need for a daytime nap, others may have outgrown the need but are still required to nap, or some children may still benefit from a nap but present behavioral challenges in compliance^8,21^. Additionally, napping patterns can be influenced by ecological factors such as family practices, parent sleep and work routines, childcare attendance, technology use^21^, and potentially movement behaviors.

Although mechanisms between sleep and physical activity in young children has not been explored as in adults^11^, it is possible that napping may help children feel more restored and energized for subsequent movement activities. Additionally, in a sample of primary school children in China (where daily napping as part of the school schedule is prevalent), the authors noted positive associations between nap frequency and duration with several cognitive, psychological, and behavioral health outcomes^22^. Thus, as both nap sleep and movement behaviors appear to have favorable relations with similar health measures^8,22^, there may be a link between these behaviors.

Among research regarding movement behaviors and nap sleep in young children, studies are limited and primarily observational. Although findings in two infants studies do not suggest a relation between movement behaviors and sleep^23,24^, the preschool years reflect a transitional period for both domains. In addition to the sleep changes noted above (e.g., decreases in nap frequency), children begin participating in more MVPA across the later early childhood years^21,25^. Consequently, relations between wake and sleep behaviors could evolve as well. In a sample of preschool children with parent-reported measures, Yu et al.^26^ did not observe an association between physical activity and daytime sleep. However, in one of our recent studies with accelerometry-measured behaviors, we demonstrated with compositional data analysis that theoretical time reallocations of some 24-h behaviors (i.e., estimating effects on an outcome by adding time to sedentary time, light physical activity, MVPA, and sleep at the expense of one another) had significant estimated effects on nap frequency^27^. For example, reallocating time away from sedentary behaviors or light physical activity was associated with an increase in nap frequency. To further explore the role of nap frequency in the current study, our first aim was to determine if movement behaviors differ between habitually napping and non-habitually napping children.

Polysomnography (PSG) is considered the gold standard of sleep measurement that can derive more measures than accelerometry (e.g., sleep staging), yet it has had limited use in youth studies in relation to movement behaviors. Characteristics of EEG, EMG, and EOG during sleep allow for the identification of sleep stages from PSG including non-rapid eye movement stages 1–3 (N1, N2, and N3) and rapid-eye movement sleep (R). Exercise and physical activity are related to PSG measured N2 and N3 sleep in adults^11,28^ and older children^29–34^. However, to our knowledge sleep physiology has not been examined in relation to movement behaviors in early childhood^35,36^ samples. Thus, our second aim was to determine if movement behaviors are associated with nap sleep architecture and spindles in N2 sleep.

Experimental study designs have demonstrated that napping can be beneficial in early childhood, although reported effects appear to vary by task domain. Napping during the preschool years has a positive effect on memory performance (i.e., declarative and procedural memory), generalization of information, and emotion processing^37,38^. This benefit is thought to reflect memory consolidation, with effects appearing either immediately or after a delayed period (e.g., the next morning). Evidence regarding the benefits of napping for other developmental, behavioral, and health factors (e.g., stress markers, other cognitive domains, accidents, and obesity-related risk factors such as physical activity and sedentary behaviors), are less clear^37,39^. Indeed, reviews have noted that studies exploring nap sleep and markers of physical health, particularly with experimental designs^14,15^, are relatively limited and few have explored movement behaviors. Additionally, it appears that, as the brain matures in early childhood, daytime naps may become less essential and nap habituality (i.e., typical nap frequency) may moderate some daytime sleep-related benefits^18^.

Similar to previous nap paradigms in early childhood, exploring the acute effects of missing one daytime nap with respect to movement behaviors may be an appropriate place to start. Missing a nap opportunity, particularly if napping is habitual for a child, may increase sleepiness and negative affect, which in turn could contribute to an increase in sedentary time and potentially an increase in light physical activity (e.g., more fidgeting throughout the day to counter potential sleepiness). Therefore, our third aim was to determine if the level of movement behaviors differ after an afternoon of nap-promotion, compared to wake-promotion, on the same day and subsequent day.

**Figure 1:**
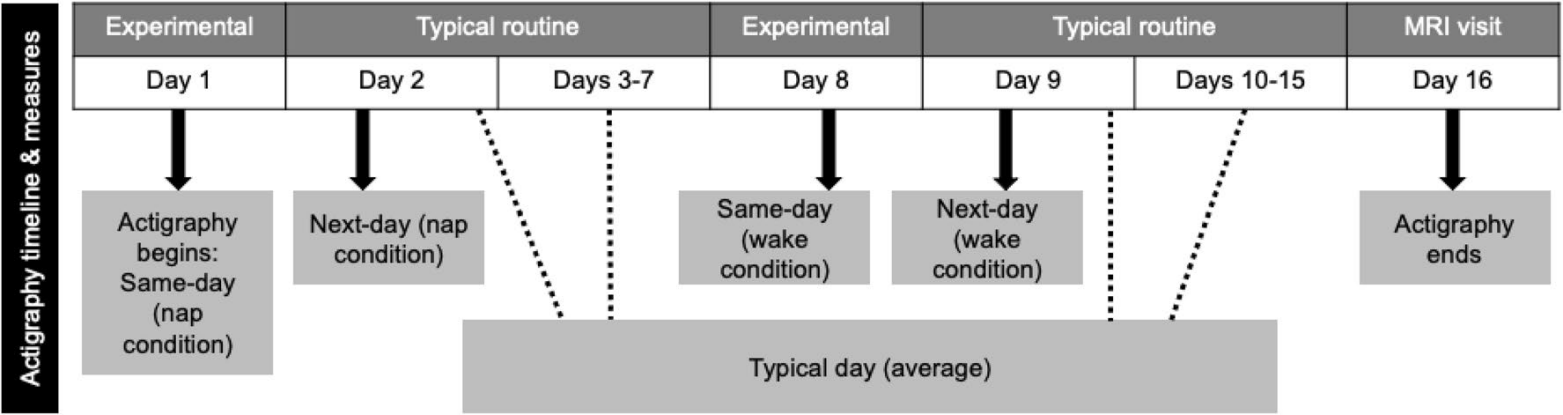
Study overview. Actigraphy was measured throughout the full period. Children followed their normal routines on non-experimental days and ‘typical’ behavior was determined from these days. On days shaded noted as experimental, children were either nap- or wake-promoted in the afternoon (order counter-balanced). Polysomnography was measured during the nap on the nap condition day. Actigraphy for each of the two conditions was determined separately for the same day of the condition (‘same’ day) and for the day immediately following the condition (‘next’ day).

Here, we explore novel questions regarding relations between napping and children’s activity during early childhood that could contribute to our knowledge about 24-h behaviors in young children, and potentially inform physical activity and sleep intervention and recommendations. To address our aims, we conducted a secondary analysis of both observational and experimental data (collected with a primary goal of examining the role of sleep and hippocampal development in memory performance during early childhood) from a study that completed data collection in the spring of 2022. At each time point of the parent study, children participated in two in-home sessions, a nap-promotion and wake-promotion (order counterbalanced) condition, approximately one week apart (Fig. 1). Actigraphy over 13 days was used to measure wake behaviors (i.e., sedentary time, light physical activity, and MVPA) on typical (days without nap- or wake-promotion conditions) and experimental days (days with nap- or wake-promotion conditions), as well as weekly nap frequency (determined from days without nap- or wake-promotion conditions). Nap physiology was measured with PSG on the nap-promotion day only.

Although our analyses were exploratory in nature given the paucity of research in early childhood exploring relations with nap sleep, we pursued the following hypotheses. First, we hypothesized that non-nappers would participate in greater MVPA compared to children that napped regularly. Second, based on the reports in older children and adults described above, we predicted that greater MVPA would be positively related to N3 sleep. Finally, we anticipated that following a day of a missed nap, children (particularly habitual nappers) would experience less MVPA that evening and on the next day.

## Results

### Participant characteristics

Participant characteristics are presented in Table 1. Forty-four participants, ranging from 3.1 to 5.8 years old, were included in one or more of the analyses. The sample consisted of 17 habitual nappers (children than typically napped 5 or more days/week), 15 intermediate nappers (2–4.9 days/week), and 11 non-nappers (less than 2 days/week). A small proportion of children napped either every day of the week (n = 2) or never (n = 4). For average movement behaviors (e.g., non-experimental days), participants wore the Actiwatches an average of 12.3 ± 3.1 days (range = 3 to 17 total days, weekdays = 8.9 ± 2.3 days, weekends = 3.3 ± 1.3 days) with 750.0 ± 52.5 min of wear time. Averages ranged from 26.4 to 60.4%, 30.6–52.1%, and 4.6–29.7% for sedentary time, light physical activity, and MVPA respectively. During the sleep-promoted nap condition with PSG measures, participants slept for an average of 88.6 min, with an average of 9.3 min in N1 sleep, 31.2 min in N2 sleep, 47.5 min in N3 sleep, and 0.6 min in R sleep.

**Table 1:** Participant characteristics.

	Full sample (n = 44)	Habitual nappers (n = 17)	Intermediate nappers (n = 15)	Non-nappers (n = 11)	*p* value*
	Mean (SD) or n (%)	Mean (SD) or n (%)	Mean (SD) or n (%)	Mean (SD) or n (%)	
**General characteristics**					
Age	4.2 (.6)	3.9 (0.4)	4.2 (0.7)	4.5 (0.8)	0.12
Female	24 (55.6)	10 (58.8)	7 (46.7)	6 (54.6)	0.48
Naps/week	3.7 (2.1)	5.6 (.7)	3.6 (1.0)	.6 (.7)	< 0.00
Race					0.33
White	28 (63.6)	10 (58.9)	10 (66.6)	8 (82.7)	
Black/AA	6 (13.6)	3 (17.6)	3 (20.0)	0	
Asian	1 (2.3)**	0	0	0	
Asian and white	3 (6.8)	0	1 (6.7)	2 (18.3)	
Unknown	6 (13.6)	4 (23.5)	1 (6.7)	1 (9.0)	
Hispanic	3 (6.8)	0	2 (13.3)	1 (9.0)	0.36
Parent education					0.65
High school	2 (4.5)	1 (5.9)	1 (6.7)	0	
Some college	1 (2.2)	0	1 (6.7)	0	
4-yr college	5 (11.4)	1 (5.9)	3 (20.0)	1 (9.1)	
Post-graduate	31 (70.5)	12 (70.6)	9 (59.9)	9 (82.2)	
Unknown	5 (11.4)	3 (17.6)	1 (6.7)	1 (9.1)	
Annual household income					0.12
< $15,000	1 (2.2)	1 (5.9)	1 (6.7)	0	
$15,000–35,000	2 (4.6)	1 (5.9)	0	0	
$35,000–55,000	3 (6.8)	0	3 (20.0)	0	
$55,000–$75,000	0	0	0	0	
$75,000–$115,000	4 (9.1)	1 (5.9)	3 (20.0)	0	
> $150,000	34 (77.3)	14 (82.3)	8 (53.3)	11 (100)	
Time point					0.002
Baseline	22 (50.0)	12 (70.6)	9 (60.0)	1 (9.2)	
6-months	16 (36.4)	5 (29.4)	5 (33.3)	5 (45.4)	
12-months	6 (13.6)	0	1 (6.7)	5 (45.5)	
**Typical movement behaviors during wake**					
Sedentary time (%)	41.2 (7.6)	42.4 (7.4)	41.2 (7.4)	40.4 (8.8)	0.79
Light PA (%)	41.8 (7.6)	41.5 (4.5)	41.6 (5.2)	42.3 (3.4)	0.91
MVPA (%)	17.0 (5.0)	16.1 (4.8)	17.1 (5.2)	18.5 (5.4)	0.46
Total PA (counts/min)	599.31 (105.44)	577.9 (98.0)	601.2 (98.1)	628.9 (5.4)	0.47
**Nap sleep physiology**					
Sleep duration (min)	88.6 (20.4)	96.1 (14.2)	89.7 (23.2)	74.7 (20.7)	0.03
N1 sleep (%)	10.5 (8.8)	6.1 (14.2)	11.9 (23.2)	11.2 (20.7)	0.71
N2 sleep (%)	34.9 (9.3)	33.9 (15.2)	33.6 (13.4)	38.1 (9.3)	0.70
N3 sleep (%)	51.7 (13.8)	52.7 (16.9)	50.8 (12.5)	50.7 (11.3)	0.91
R sleep (%)	0.6 (2.1)	0.8 (2.6)	1.0 (2.3)	0 (0)	0.51
N2 spindles (#)	14.2 (12.6)	10.3 (7.6)	17.1 (18.1)	16.8 (9.6)	0.28
N3 spindles (#)	9.3 (11.3)	7.3 (8.0)	8.0 (13.0)	14.0 (15.1)	0.66
N2 spindle density (ratio)	0.51 (0.51)	0.35 (0.33)	0.52 (0.41)	0.78 (0.78)	0.12

*Differences in characteristics between nap habit groups (one-way ANOVA models for continuous variables, chi-square tests for categorical variables).

**Data for nap habituality was missing for this participant.

### Movement behaviors by nap habituality

**Figure 2:**
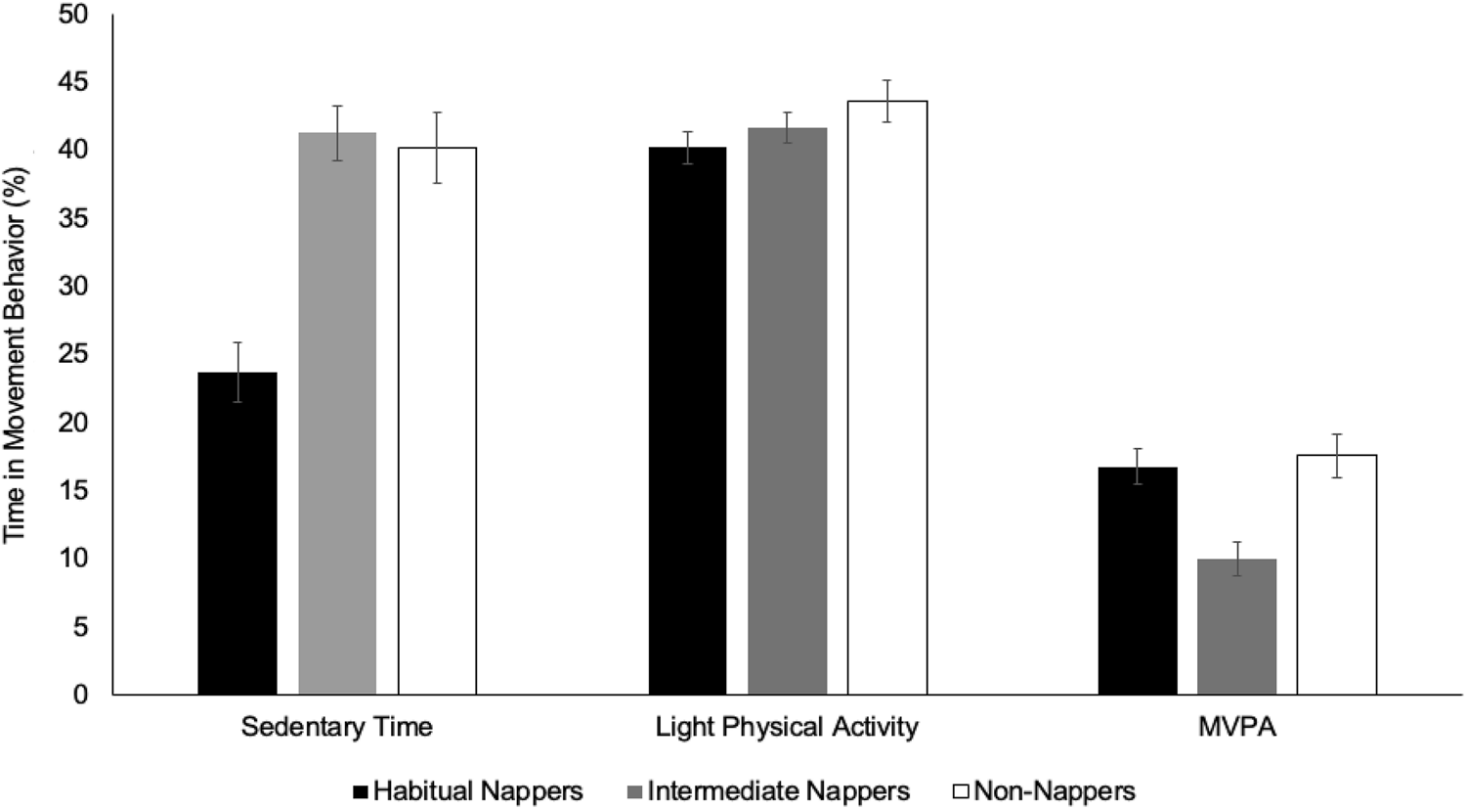
Adjusted means and standard errors of habitual wake behaviors by nap habituality group. (*MVPA* moderate- to vigorous-intensity physical activity).

To examine if movement behaviors differed by nap habitually group, we used ANCOVA models adjusted for age and average overnight sleep duration (n = 3 models). Non-nappers spent less time sedentary and more time in light physical activity and MVPA than habitual and intermediate nappers on typical days (i.e., average behaviors on non-experimental days). However, none of the average movement behaviors differed significantly between nap habituality groups (sedentary time: F(4, 37) = 0.60, *p* = 0.67; light physical activity: F(2, 37) = 1.18, *p* = 0.34; MVPA: F(4, 37) = 2.21, *p* = 0.08; Fig. 2).

### Associations between movement behaviors and nap sleep stages

To determine if wake behaviors were associated with nap sleep stages and physiology, we ran separate multiple linear regression models with each of the N nap sleep stages (i.e., N1, N2, and N3) and microstructural variables (i.e., total sleep spindles in N2, and N2 spindle density) as dependent variables (Table 2). Age, nap group, and an interaction of movement behavior X nap group were explored as covariates. Given our sample size and that only the addition of age appeared to increase the amount variance explained by each model, we elected to use the more parsimonious models that adjusted only for age. Given stage R sleep is often not a component of nap sleep in preschool-aged children^40–42^, and that the majority of the sample had little to no R sleep, we did not explore that stage in our analyses. Movement behaviors were not associated with nap sleep stages or microstructural variables. Higher levels of sedentary time were associated with a greater proportion of nap sleep spent in the N1 and N2 stages, and less time in N3. However, these correlations were not statistically significant (all *p*’s > 0.31). Light physical activity and MVPA followed similar patterns such that less time spent in these behaviors was associated with more N1 and N2 nap sleep, whereas more time in these behaviors was associated with more N3 sleep. However, again these associations were not statistically significant (all *p*’s > 0.21).

**Table 2:** Linear regression results for associations between movement behaviors and nap sleep stages.

Sleep physiology measures	Movement behaviors								
	Sedentary time			Light PA			MVPA		
	Coef	F (df)	95% CI	Coef	F (df)	95% CI	Coef	F (df)	95% CI
N1 stage	0.12	2.0 (2, 38)	− 0.24, 49	− 0.32	2.3 (2, 38)	− 0.9, 0.31	− 0.080	1.8 (2, 38)	− 0.62, 0.46
N2 stage	0.25	1.3 (2,38)	− 0.32, 0.83	− 0.59	1.6 (2,38)	− 1.6, 0.41	− 0.02	0.8 (2, 38)	− 0.89, 0.85
N3 stage	− 0.29	2.4 (2,38)	− 0.84, .28	0.61	2.8 (2,38)	− 0.36, 1.6	0.16	1.9 (2,38)	− 0.69, 1.0
N2 spindle #	0.09	2.2 (2,38)	− 0.42, .61	0.32	2.4 (2,38)	− 0.57, 1.2	− 0.49	3.1 (2,38)	− 1.2, 0.36
N2 Spindle Density	− 0.004	1.8 (2,38)	− 0.03, 0.02	0.03	2.8 (2,38)	− 0.01, 0.06	− 0.009	1.8 (2,38)	− 0.04, 0.02

Linear regression models were adjusted for age. *df* degrees of freedom, *CI* confidence intervals, *PA* physical activity, *MVPA* moderate- to vigorous-intensity physical activity.

### Movement behaviors on nap- and wake-promotion days

**Figure 3:**
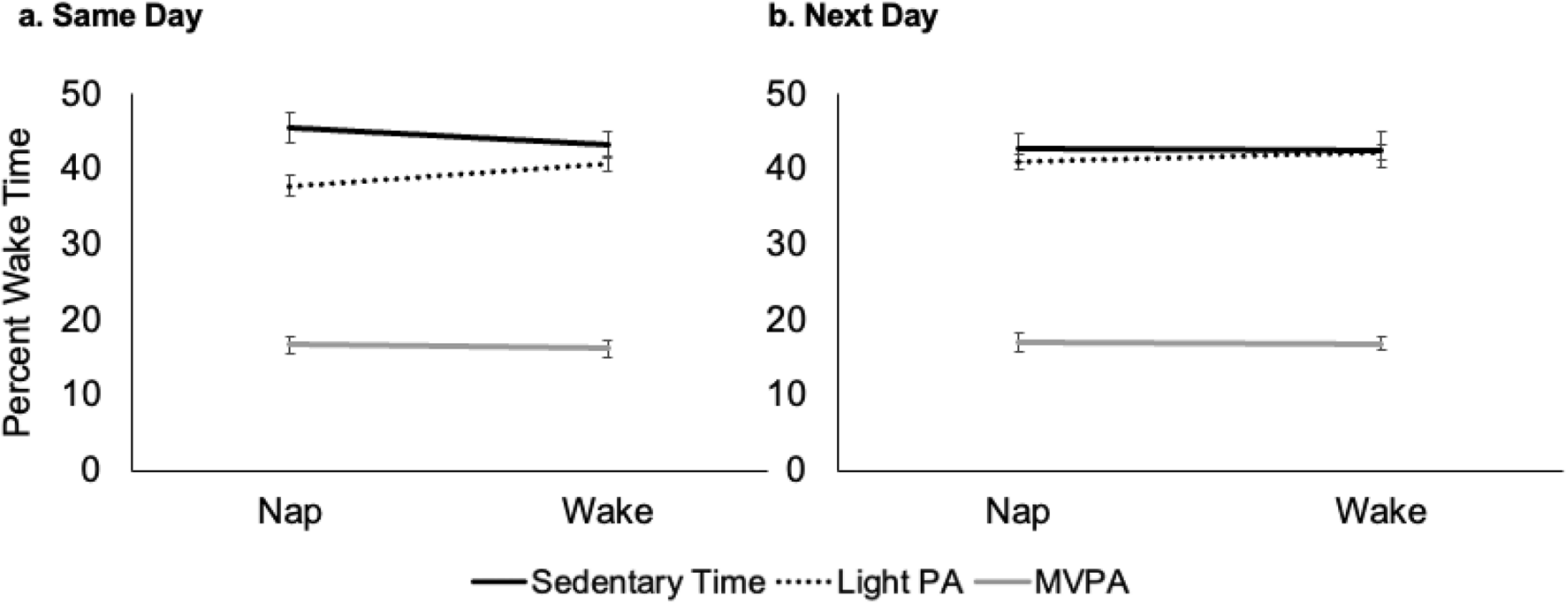
Predicted means and standard errors of wake behaviors by nap group on a) experimental condition days and b) the next day following each experimental day. (*Light PA* light intensity physical activity, *MVPA* moderate- to vigorous-intensity physical activity).

Finally, repeated measures mixed models (n = 6 in total) with condition (nap or wake promotion) as the within-subject factor were explored to determine if the level of movement behaviors differed after an afternoon of nap-promotion, compared to wake-promotion, on the same day and the subsequent day. Initially, age, nap group, overnight sleep, and an interaction of movement behavior X nap group were explored as covariates. Final models were adjusted for age and average overnight sleep duration (as overnight sleep duration of the specific night prior to the experimental day was not available for both conditions). There were no significant main effects of condition (i.e., nap- vs wake-promotion) on any of the movement behaviors on the same day of the experimental condition (Fig. 3a; Table 3). We also explored whether movement behaviors on the mornings of the nap- or wake-promotion conditions differed between days, as this could also influence behaviors following the conditions. However, there were no differences in sedentary time, light physical activity, MVPA or total activity between mornings (Supplemental Table 1).

**Table 3:** Repeated measures mixed model results for models exploring effects of nap- and wake-promotion on same day movement behaviors.

	Coefficient	SE	z	*p* value	95% CI	
**Model 1: same day sedentary time**						
Wake condition	− 2.28	2.4	− 0.95	0.343	− 6.98	2.42
Age	2.8	2.0	1.38	0.168	− 1.2	6.8
Night sleep duration	− 0.03	0.01	− 2.21	0.027	− 0.05	− 0.003
Intercept	47.72	9.35	5.11	< 0.001	29.4	66.03
**Model 2: same day light physical activity**						
Wake condition	2.83	1.63	1.74	0.082	− 0.36	6.03
Age	− 2.3	1.3	− 1.81	0.070	− 4.8	− 0.2
Night sleep duration	0.006	0.007	0.82	0.413	− 0.008	0.02
Intercept	49.33	5.86	7.57	< 0.001	32.85	55.82
**Model 3: same day MVPA**						
Wake condition	− 0.42	1.45	− 0.29	0.772	− 3.26	2.42
Age	− 0.47	1.39	− 0.34	0.736	− 3.18	2.25
Night sleep duration	0.02	0.008	2.71	0.007	0.006	0.04
Intercept	7.04	6.33	1.11	0.266	− 5.36	19.45

*SE* standard error, *CI* confidence interval, *MVPA* moderate- to vigorous-intensity physical activity.

### Movement behaviors on days following nap- and wake-promotion

We ran similar repeated measures mixed models (n = 6 in total) to explore next-day movement behaviors, but in addition to age, models were adjusted for sleep duration of the experimental night rather than mean sleep duration. Comparable to the same day results, there were no significant main effects of condition (i.e., nap- vs wake-promotion) on any of the movement behaviors on the day immediately following each of the experimental conditions (Fig. 3b; Table 4).

**Table 4:** Repeated measures mixed model results for models exploring effects of nap- and wake-promotion on next day movement behaviors.

	Coefficient	SE	z	*p* value	95% CI	
**Model 1: next day sedentary time**						
Wake condition	1.3	1.96	0.66	0.508	− 2.55	5.15
Age	− 1.7	2.8	− 0.65	0.517	− 7.18	3.61
Prior night sleep dur	− 0.007	0.01	− 0.63	0.530	− 0.03	0.02
Intercept	51.92	11.54	4.5	< 0.001	29.29	74.55
**Model 2: next day light physical activity**						
Wake condition	− 0.31	1.17	− 0.27	0.790	− 2.61	1.98
Age	− 0.8	1.3	− 0.6	0.547	− 3.4	1.8
Prior night sleep dur	0.0008	0.005	0.16	0.875	− 0.009	0.01
Intercept	45.7	5.49	8.32	< 0.001	34.97	56.51
**Model 3: next day MVPA**						
Wake condition	− 0.19	0.97	− 0.2	0.842	− 2.09	1.7
Age	− 1.26	1.52	− 0.83	0.407	− 4.2	1.7
Prior night sleep dur	0.01	0.005	1.93	0.054	− 0.0002	0.02
Intercept	17.1	6.17	2.77	0.006	5.02	29.19

*SE* standard error, *CI* confidence interval, *MVPA* moderate- to vigorous-intensity physical activity.

## Discussion

Although daytime napping is common in early childhood and appears to facilitate learning, benefits may be modified by developmental maturation and are less clear for physical health-related behaviors such as time spent sedentary and active. In the present study, we leveraged data from an experimental study of napping in preschool children to understand how naps interact with movement behaviors. In our sample of preschool children, time spent in movement behaviors did not significantly vary by how often children typically napped and were not associated with nap sleep stages. Moreover, movement behaviors did not differ following a day with a nap compared to a day without a nap, regardless of nap habituality.

As expected, age correlated with nap frequency in our sample and children that were considered non-nappers engaged in more light physical activity and MPVA and less sedentary time relative to habitual and intermediate nappers. However, differences in movement behaviors when participants were grouped by typical weekly frequency of napping were not significant. In our sample, sedentary time was slightly lower, and light physical activity and MVPA were slightly higher in non-nappers relative to regular and intermediate nappers, although these differences were not statistically significant. One consideration when interpreting these null findings is the lack of information regarding the reason for napping (or not napping) in our sample. It is possible that some children that do not nap frequently may still benefit from a regular nap and conversely, some children may no longer have a physiologic need, but napping is still part of their routine.

Comparable to our null findings, two previous studies in infants^23,24^ and one in preschoolers^26^ reported no association between nap frequency or sleep with sedentary time or physical activity. In the present study, we did not consider the reason for napping. For example, some children may still benefit from a nap but could be considered non-nappers due to behavioral challenges and trouble transitioning to nap opportunities^18^. While future studies may want to consider the reason for napping, it is also possible as suggested by our current findings, that in generally healthy young sleepers, nap frequency is not an influential factor on movement behaviors. However, we cannot rule out the possibility of residual confounding from unmeasured factors (e.g., food intake^8,43^).

Studies exploring sleep physiology and movement behaviors or related measures (e.g., sports participation or fitness) in youth are relatively limited, but overall findings have been mixed and PSG measures have been for overnight sleep. Findings from observational studies in adolescents have reported a positive association between high intensity sport participation and deep sleep^31^, a positive association between total estimated energy expenditure and N2 sleep^29^, and a null association between both questionnaire-reported and accelerometry-measured physical activity and any sleep stage^30^. Experimental studies in older children and adolescents have demonstrated some preliminary evidence of both acute and chronic structured activity and N3 sleep^32,44^.

In the current study of early childhood however, no significant associations between movement behaviors and nap sleep stages were observed. In addition to teasing apart moderate and vigorous activities, contextual and timing information of movement behaviors may be important aspects to consider in young children to better understand if similar effects are present as reported in older children and adolescents. Additionally, our predictions were based on available studies which are limited to overnight sleep and the consideration that percent time in nap sleep stages appears to stay consistent across early childhood^40^. However, nap sleep physiology differs a bit from overnight sleep. For example, naps typically contain little to no R sleep^40–42^. Differences in sleep architecture could possibly contribute to the contrasting associations observed here from findings in adults and older youth using overnight sleep. Furthermore, in the current study we explored nap sleep stages and N2 spindle frequency as markers of sleep physiology, but future work may consider including other metrics such as frequency bands i.e., (slow oscillations, delta, theta, sigma).

Although one day of a missed nap opportunity may not result in substantial sleep loss in young children, our predictions were based on available studies investigating sleep restriction or poor sleep in adolescents and older adults. While many studies have focused on the role that movement behaviors play on sleep, sleep loss can also decrease physical performance and alter movement behavior patterns^11–13^. Supported mechanisms between acute sleep loss and performance include changes in metabolism (i.e., increased insulin resistance) and increased biomarkers of inflammation which could decrease heat tolerance and possibly be linked to decreased alertness and increased perceived exertion during activity^11^. However, these pathways have not been adequately explored in young children.

In the present study, missing a nap did not appear to influence subsequent wake behaviors, even among children that napped habitually. Similarly, when we recently explored within-person temporal associations between overnight sleep duration and next day wake behaviors with an observational design, when children slept more or less overnight relative to their personal average (or ‘typical’ night), this did not appear to be associated with their next-day behaviors^45^. It is possible that one afternoon of nap sleep restriction may not be unusual for this age group. Indeed, only a small proportion typically napped every day of the week (i.e., 4.8%). Additional days of missed naps may have different effects and is something future research could explore.

Strengths of the current study include the use of objective measures of wake and sleep (i.e., device- and PSG-measured) behaviors and the study design. Most early childhood studies have used only parent-report or actigraphy, so the addition of PSG was novel and allowed us to look at objective sleep structure metrics. Additionally, our study builds on previous observational work with an experimental component (i.e., the manipulation of nap versus wake).

However, some limitations should be noted. Generalizability may be limited as our sample was generally healthy. Participants were likely to be good sleepers given the eligibility criteria. Additionally, the overall socioeconomic status of the sample was high, which has been linked to both physical activity and sleep health^8,18^. Moreover, although morning movement behaviors of the nap- and wake-promotion days did not differ, we cannot completely rule out the influence of morning activity levels given that only a small sample (n = 8) had actigraphy data on both days for those time intervals. Although overnight sleep duration did differ on the nap- and wake-promotion nights, due to sample size, we did not add this measure as a covariate to our models exploring next-day movement behaviors. However, as indicated by one of our recent studies of daily associations^45^, a single night of longer or shorter sleep may not pose a meaningful influence on next day movement behaviors in preschool children. Finally, our study may have been underpowered for small to moderate effects of nap sleep on same and next day movement behaviors.

Findings from our study do not indicate relations between movement behaviors and nap habituality or nap sleep stages in healthy preschool-aged children. Additionally, a single missed nap opportunity did not appear to influence the proportion of time spent in sedentary or in physically active behaviors on the same or subsequent day. However, despite the null findings, the benefits of nap sleep on movement behaviors should not be entirely ruled out and future research can extend our initial analyses to better inform our understanding of these important health behaviors in early childhood. Specifically, we recommend that researchers examine extending our protocol to multiple missed nap opportunities (or multiple nap opportunities for children that do not habitually nap) in a larger, diverse (e.g., health and socioeconomic status) sample.

## Methods

All research involved in this investigation was in compliance with the guidelines and regulations in accordance to the Declaration of Helsinki. The study protocols were approved by the University of Maryland Institutional Review Board. Adult caregivers provided written informed consent, and verbal assent was obtained from child participants.

### Participants

Participants were recruited from the University of Maryland Infant and Child Studies database, and through word of mouth, campus and community flyer postings, and email messages on related listservs. Inclusion criteria of the parent study included: 3–6 years of age, habitually napping (based on parent report, naps 5–7 days/week), native-English speaking, normal or corrected-to-normal vision, normal or corrected-to-normal hearing, no current or past diagnosis of a developmental disability or sleep disorder, no use of psychotropic or sleep-affecting medications, no fever or respiratory illness at the time of testing, no recent travel outside of the local time zone, no history of neurological injury, and no presence of metal in the body (as the parent study included MRI).

**Figure 4:**
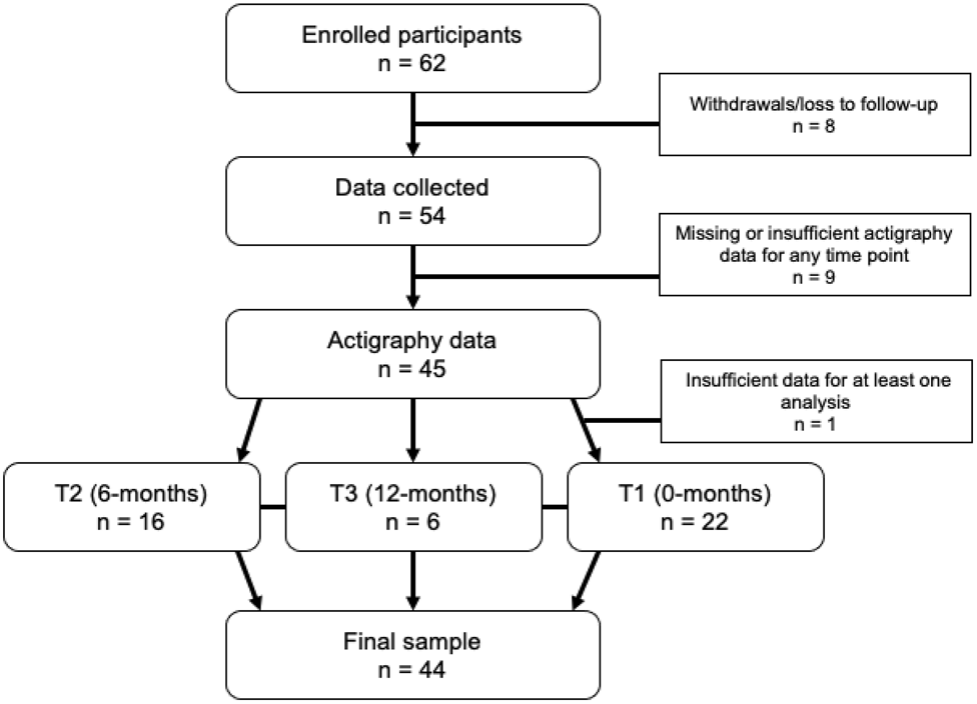
Participant flow diagram. (*Note* Data were pooled from the 6-month, 12-month, and then baseline time points, respectively, *T* time point).

Data for the parent study was collected at three different time points (time point 1 [T1]; time point 2 [T2] = 6 months after T1; and time point 3 [T3] = 12 months after T1). For the current study, data from one time point for each participant was selected and pooled together. An aim of the parent study was to capture children as they transitioned out of regular napping, and thus most participants were habitual nappers at T1. Given that one of our objectives was to assess nap habituality as a moderator, we prioritized data at T2 as that time point had the most variability in nap habituality (i.e., a distribution of habitual nappers, intermediate nappers, and non-nappers). To be included in the present study, participants needed to have at least 3 days of 480 min of wear time (see Movement Behaviors in Measures section for more details) during wake intervals on typical (i.e., non-experimental) days. Additionally, participants needed at least one of the additional measures of interest to our research questions (i.e., same-day actigraphy for both nap-promotion and wake-promotion, next-day actigraphy for both nap-promotion and wake-promotion, or PSG from the nap-promotion condition). If a participant was missing one or more of these additional measures at T2, then data from T3 or T1 were used if those time points had more complete data (Fig. 4).

### Protocol

In the first session, parents were given a sleep diary, an electronic link to a questionnaire, and actigraphy instructions. Additionally, children were given an actigraphy watch and asked to wear it constantly (i.e., 24-h/day) for approximately 13 days. In the nap-promotion session, research staff and parents encouraged children to sleep by providing verbal encouragement, back-rubbing, and soothing techniques. Approximately 30–45 min prior to the start of the child’s typical nap time, children were equipped with PSG. The equipment was removed when the participant woke from the nap. To promote wake in the wake-promotion session, methods such as verbal encouragement and quiet activities (e.g., book reading and puzzles) were used.

### Measures

#### Movement behaviors

Movement behaviors during daytime wake intervals were derived from Actiwatch Spectrum (Philips Respironics, Bend, OR) actigraphy monitors that were worn on the non-dominant wrist. The Actiwatch is a triaxial and water-resistant accelerometer that provides off-wrist detection and is equipped with an event marker button. Parents and children were instructed to press the event marker button to note lights on and lights off (as an indicator of the beginning and end of the sleep opportunity). Validity of this device to estimate daytime energy expenditure was explored in a sample of preadolescent children (mean age = 8.8 years), with indirect calorimetry as a comparison (r = 0.90, *p* < 0.001)^46^. From these data, Ekblom et al. also proposed activity count thresholds for physical activity intensities for each 15-s epoch (i.e., sedentary ≤ 79, light intensity physical activity = 80–261, MVPA ≥ 262 counts). Intra- and inter-instrument coefficients of variation ranging from 0.72 to 8.4% demonstrated the reliability from this tool when tested in a mechanical shaker. In a sample of preschool children, Alhassan et al.^47^ subsequently conducted a cross-validation study using direct observation as a criterion comparison to the Ekblom et al. cut points (r_sp_ = 0.47, *p* < 0.001).

In the present study, Actiwatches (sampling rate = 32 Hz and sensitivity < 0.01) collected data in 15-s epochs and data were downloaded in the Actiware software (Philips Respironics, Bend, OR). Using a combination of sleep diaries and event marker button presses, rest (i.e., time in bed) intervals were identified. When neither diaries or event markers were present, sleep onset was defined as the first 3 consecutive minutes of sleep and sleep offset was defined as the last 5 consecutive minutes of sleep. All epochs were then scored using Actiware’s default algorithm^48^, which has been validated in preschool samples^49,50^, and classified each epoch as rest (i.e., wake during time in bed), sleep (sleep during time in bed), or wake (wake during time out of bed and daytime).

Epochs defined as wake were then processed to calculate the movement behavior variables (i.e., total activity and percent time sedentary, in light physical activity, and in MVPA) for: (1) the average of non-experimental condition days—referred to as typical movement behaviors, (2) the two days with experimental conditions (i.e., day of nap-promotion and day of wake-promotion), (3) the next day following the two experimental conditions (i.e., day following nap-promotion and day following wake-promotion). Although a daily minimum daytime wear time during wake of 600 min has been suggested for this age group for accelerometry^51^, given that most of our sample had sleep of 60–120 min within the daytime interval, a minimum of 480 min of wake were set as the criteria for wake behavior variable processing. Additionally, we included only participants with at least three days of sufficient wear time during wake for the typical daily average variable. Total activity was parameterized as mean daily activity counts/min, calculated as the sum of the wake activity epoch counts each day and divided by the wake wear time for that day. Daily percent time spent sedentary, in light physical activity, and in MVPA were calculated from the Ekblom et al.^46^ cut points.

#### Nap sleep stages and physiology

The Embletta MPR (Natus) ambulatory PSG system with 14-electrode montage, was used for PSG using the 10–20 system, to assess nap sleep stages. The montage consisted of two EOG leads, two chin EMG leads, and 10 cortical EEG leads (F3, F4, C3, C4, CZ, O1, O2, M1, M2, Ground), with electrodes referenced to Cz. The American Academy of Sleep Medicine guidelines for sleep scoring were followed to identify sleep stages^52^. Nap sleep stage variables included percent time in N1, N2, N3 and R sleep, as well as spindle number in N2 and N3, and spindle density in N2.

#### Demographic information and covariates

Demographic characteristics (i.e., age, sex, race, ethnicity, and socioeconomic measures) of the sample were collected via a health and demographic questionnaire completed by parents. Nap habituality was derived from actigraphy when available and calculated as the number of naps divided by the number of nap opportunities (excluding the experimental condition days) × 7. When actigraphy was not available, these data were derived from the sleep diary (n = 1) or parent questionnaire (n = 2), in that respective order. Children were classified into nap habituality groups based on nap frequency on non-experimental days and categorized as habitual nappers, intermediate nappers, or non-nappers. Actigraphy derived overnight sleep duration (minutes) was also processed for the following intervals: the average for the full study period, the night prior the nap promotion, the night prior to wake promotion night, the night of nap promotion, and the night of wake promotion). Additionally, wake behaviors (i.e., total activity and percent time sedentary, in light physical activity, and in MVPA) were calculated for the morning (i.e., the intervals defined as daytime wake from 12:00 am to 12:00 pm) of each of the experimental conditions (nap and wake promotion).

### Analysis

All analyses were conducted in Stata (Version 17.0, StataCorp LLC, College Station, TX) with an alpha level of 0.05. Descriptive analyses were run to calculate participant characteristics and differences in some covariate measures between experimental condition days or nights. Though to our knowledge, there are no published studies using a similar protocol and measures, we opted to target a moderate effect size and ran our power analyses for the repeated mixed models in G*Power^53^. For a moderate effect size (n^2^ = 0.06), 80% power, and alpha of 0.05, a sample size of 52 participants was needed. Assumptions for the linear regression and mixed models (i.e., normality, homoscedasticity, and independence of residuals) were met.

## Supplementary Information

Supplementary Table S1.

## Data Availability

The datasets used and analyzed during the current study available from the corresponding author on reasonable request.
