# Supplementary material for: Influence of naps on sedentary time and physical activity in early childhood
Source: Sci Rep. 2022 Dec 8;12:21198. doi: 10.1038/s41598-022-25628-x (PMC9731956; doi:10.1038/s41598-022-25628-x)
Supplement: Supplementary file 1 — Supplementary Table S1. [file 41598_2022_25628_MOESM1_ESM.docx]

**Supplemental Table 1.** Paired t-test results exploring differences in overnight sleep duration and morning movement behaviors between nap- and wake-condition days.

| **Measures** | **Nap-Condition**  **Mean (SE)** | **Wake-Condition**  **Mean (SE)** | **T** | **p-value** |
| --- | --- | --- | --- | --- |
| Sedentary time (%): morning of condition* | 40.2 (3.9) | 46.4 (4.8) | -0.90 | 0.398 |
| Light PA (%): morning of condition* | 41.7 (3.3) | 36.3 (3.8) | 1.14 | 0.291 |
| MVPA (%): morning of condition* | 18.2 (3.3) | 17.3 (3.3) | 0.18 | 0.864 |
| Sleep duration (min): night of condition | 495.9 (30.5) | 531.3 (38.9) | -2.86 | 0.009 |

*Only 8 participants had morning measures data for both conditions. Note: PA = physical activity; MVPA = moderate- to vigorous-intensity PA
